# Tolerance levels of CT number to electron density table for photon beam in radiotherapy treatment planning system

**DOI:** 10.1002/acm2.12226

**Published:** 2017-11-20

**Authors:** Minoru Nakao, Shuichi Ozawa, Kiyoshi Yamada, Katsunori Yogo, Fumika Hosono, Masahiro Hayata, Akito Saito, Kentaro Miki, Takeo Nakashima, Yusuke Ochi, Daisuke Kawahara, Yoshiharu Morimoto, Toru Yoshizaki, Hiroshige Nozaki, Kosaku Habara, Yasushi Nagata

**Affiliations:** ^1^ Hiroshima High‐Precision Radiotherapy Cancer Center Hiroshima Japan; ^2^ Department of Radiation Oncology Institute of Biomedical & Health Science Hiroshima University Hiroshima Japan; ^3^ Radiation Therapy Section Department of Clinical Support Hiroshima University Hospital Hiroshima Japan; ^4^ Department of Radiology Hiroshima Prefectural Hospital Hiroshima Japan; ^5^ Radiation Therapy Department Hiroshima City Hiroshima Citizens Hospital Hiroshima Japan; ^6^ Division of Radiology Hiroshima Red Cross Hospital & Atomic‐bomb Survivors Hospital Hiroshima Japan

**Keywords:** CT number, electron density, flattening‐filter‐free beam, quality assurance, tolerance level

## Abstract

The accuracy of computed tomography number to electron density (CT‐ED) calibration is a key component for dose calculations in an inhomogeneous medium. In a previous work, it was shown that the tolerance levels of CT‐ED calibration became stricter with an increase in tissue thickness and decrease in the effective energy of a photon beam. For the last decade, a low effective energy photon beam (e.g., flattening‐filter‐free (FFF)) has been used in clinical sites. However, its tolerance level has not been established yet. We established a relative electron density (ED) tolerance level for each tissue type with an FFF beam. The tolerance levels were calculated using the tissue maximum ratio (TMR) and each corresponding maximum tissue thickness. To determine the relative ED tolerance level, TMR data from a Varian accelerator and the adult reference computational phantom data in the International Commission on Radiological Protection publication 110 (ICRP‐110 phantom) were used in this study. The 52 tissue components of the ICRP‐110 phantom were classified by mass density as five tissues groups including lung, adipose/muscle, cartilage/spongy‐bone, cortical bone, and tooth tissue. In addition, the relative ED tolerance level of each tissue group was calculated when the relative dose error to local dose reached 2%. The relative ED tolerances of a 6 MVFFF beam for lung, adipose/muscle, and cartilage/spongy‐bone were ±0.044, ±0.022, and ±0.044, respectively. The thicknesses of the cortical bone and tooth groups were too small to define the tolerance levels. Because the tolerance levels of CT‐ED calibration are stricter with a decrease in the effective energy of the photon beam, the tolerance levels are determined by the lowest effective energy in useable beams for radiotherapy treatment planning systems.

## 1 INTRODUCTION

Computed tomography (CT) data have generally been used to calculate the dose to the body of a patient treated with a radiotherapy treatment planning system (RTPS). The natural body is complex and inhomogeneous with multiple tissue groups including lung, adipose, muscle, general organ, cartilage, bone, and tooth tissues. To calculate the appropriate radiotherapy dose for a human body, CT number to electron density (CT‐ED) calibration is generally performed with a calibration phantom with several inserted tissue substitutes.^1^ The accuracy of CT‐ED calibration is a key component for dose calculations in inhomogeneous mediums.

Kilby et al.^2^ established relative electron density (ED) tolerance levels based on the relative dose error to local dose using the tissue maximum ratio (TMR) and the maximum tissue thicknesses defined by multiple treatment plans. It was shown that the tolerance levels became stricter with an increase in tissue thickness and a decrease in the effective energy of the photon beam. For the last decade, a low effective energy photon beam (e.g., flattening‐filter‐free (FFF) beam) has been used in stereotactic body radiation therapy to treat lung or abdomen cancer. Therefore, new relative ED tolerance levels are required for a RTPS with an FFF beam.

The relative ED tolerance levels are useful to assure the suitability of the CT‐ED calibration table of planning CT and cone beam CT. The purpose of this study is to establish a relative ED tolerance level corresponding to each tissue type for a RTPS with an FFF beam. Moreover, we attempted to establish the relative ED tolerance levels based on standard tissue data and validate the relative ED tolerance levels with an adult anthropomorphic phantom.

## 2 METHODS

### 2.A Effective depth

The effective depth has been used in the inhomogeneity correction method in an RTPS.^3^ The relationship between the effective depth and dose is given by

(1) $$D(d)\propto \mathrm{TMR}(d_{\mathrm{eff}})$$

where *D* is the dose, *d* is the depth, TMR is the tissue maximum ratio, and *d*_eff_ is the effective depth. *d*_eff_ is given by

(2) $$d_{\mathrm{eff}}=\sum_{\mathrm{all}i}t_{i}\rho_{e,i}$$

where *t*_*i*_ is the thickness of tissue *i* and *ρ*_*e,i*_ is the ED of tissue *i* relative to water.

The relationship between the dose error, Δ*D*, and the error in the relative ED, Δ*ρ*_*e,i*_, are established by eqs. (1) and (2) 2 and is given by

(3) $$\Delta \rho_{e,i}=\frac{\Delta D/D}{t_{i}}\frac{\mathrm{TMR}}{\left(\frac{d\mathrm{TMR}}{d(d_{\mathrm{eff}})}\right)}$$

where Δ*D*/*D* is the relative dose error to local dose, (*d*TMR/*d*(*d*_eff_))/TMR is the gradient of TMR relative to the local TMR, and Δ*ρ*_*e,i*_ is the error in *ρ*_*e,i*_. TMR data were measured in water with a Varian TrueBeam STx (Varian Medical Systems, Palo Alto, CA, USA). (*d*TMR/*d*(*d*_eff_))/TMR was also measured for a 10 cm × 10 cm field at a 10‐cm depth. The relative dose error to local dose (Δ*D*/*D*) was set as 2%.^4^

### 2.B Effective tissue thickness

Tissue thickness was required to estimate the dose error caused by the CT‐ED calibration. We defined the effective tissue thicknesses using the adult reference computational phantom data (V1.2) in the International Commission on Radiological Protection publication 110 (ICRP‐110).^5^ The effective tissue thicknesses were reasonable to determine the relative ED tolerance levels.

The anthropomorphic voxel phantoms are consist of 52 standard tissues and air. The 52 tissues were classified by mass density into five tissues groups including lung, adipose/muscle, cartilage/spongy‐bone, cortical bone, and tooth tissues. The mass density border between lung tissue and adipose tissue is *ρ *= 0.90 g cm^−3^.^6^ Only lung tissue had a mass density below the border value of *ρ *= 0.90 g cm^−3^, while adipose, muscle, general organ, and some spongy‐bone tissues had values between 0.90 g cm^−3^ and 1.07 g cm^−3^ (Male: cervical spine, sternum, and sacrum, Female: sacrum and femora). Skin, cartilage, and most spongy‐bone tissues had values between 1.07 g cm^−3^ and 1.25 g cm^−3^. Furthermore, cortical bone tissue had a value of 1.92 g cm^−3^, and tooth tissue had a value of 2.75 g cm^−3^. The maximum thicknesses of the classified tissue groups were measured from axial plane to define the corresponding effective tissue thickness.

### 2.C Relative ED tolerance level

The relative ED tolerance levels were generated using eq. (3) and the effective tissue thicknesses of each tissue group. The TMR of the 6 MVFFF beam was used to estimate tolerance levels because the effective energy is the lowest of the five photon beams and because the beam was used against deep tumours more frequently than the 4 MV beam.

### 2.D CT number constancy

CT number constancy was required to evaluate the relative ED tolerance levels. A Catphan 700 phantom (The Phantom Laboratory, Salem, NY, USA) scanned with a CT scanner (GE Optima 580, GE Medical Systems) for routine quality assurance over a 20‐month period. For this study, a geometry and sensitometry module (CTP682) was used.

### 2.E Treatment planning with an adult anthropomorphic phantom

To validate the relative ED tolerance levels for clinical situations, the dose error caused by relative ED errors was validated in several typical treatment plans. The female adult anthropomorphic phantoms (ATOM dosimetry phantom 702‐T and 702‐P, Computerized Imaging Reference Systems, Inc., Norfolk, Virginia, USA) were scanned with a CT scanner (GE Optima 580, GE Medical Systems), and CT images were imported into the Eclipse treatment planning system (Varian Medical Systems, Palo Alto, CA, USA). Two spherical clinical target volumes (CTV) were contoured at the centre of the lung and pelvis. Planning target volumes (PTV) were contoured by adding an 8 mm CTV–PTV margin.

The dose calculation algorithm was an anisotropic analytical algorithm. Initial dose calculations were implemented with two kinds of energies and two kinds of delivery techniques for the two sites (lung and pelvis) including: two‐three‐dimensional conformal radiotherapy (3DCRT) plans for lung tumours (6 MV and 6 MVFFF, 2 fields and 8 fields), and two 3DCRT plans for pelvic tumours (10 MV and 10 MVFFF, 2 field and 4 fields).

The beam orientations of 2 field plans were anterior‐posterior (AP) and left‐right (LR) for lung tumours and pelvic tumours, respectively. Following the initial dose calculation, a secondary dose calculation including a CT‐ED calibration error was implemented by increasing the dose to be equal to the relative ED tolerance levels of the 6 MVFFF beam. The CT‐ED calibration error was added to the initial CT‐ED calibration table by each tissue types. The dose error between the initial and secondary calculated doses was validated by point doses at the isocentre and the CTV mean doses with the same beam parameters.

## 3 RESULTS

### 3.A Tissue thickness and relative ED tolerance

**Figure 1:**
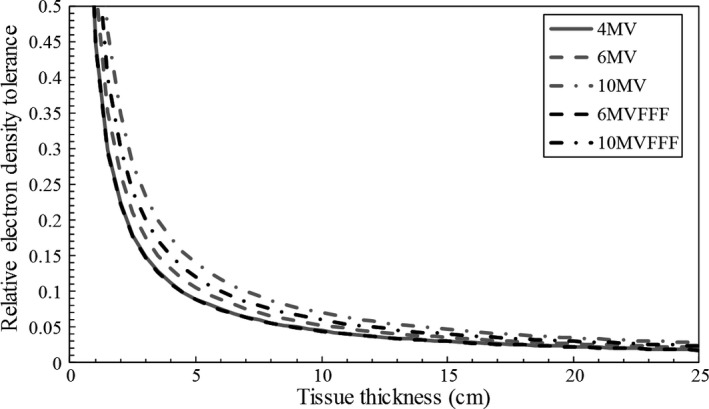
The relative electron density tolerance regarding each energy and tissue thickness for a relative dose error of 2%. Lines of 4 MV beam and 6 MV flattening‐filter‐free (FFF) beam overlap.

The relationship between tissue thickness and relative ED tolerance was described by eq. (3). The gradients of TMR, which were measured for a 10 × 10 cm field at a 10‐cm depth, were 3.3% cm^−1^ (4 MV), 2.9% cm^−1^ (6 MV), 2.4% cm^−1^ (10 MV), 3.3% cm^−1^ (6 MVFFF), and 2.7% cm^−1^ (10 MVFFF). Figure 1 shows the relative ED tolerances corresponding to each energy and tissue thickness for a relative dose error of 2%. The relative ED tolerances at 10 cm tissue thickness were 0.045, 0.053, 0.070, 0.044, and 0.060 for 4 MV, 6 MV, 10 MV, 6 MVFFF, and 10 MVFFF, respectively.

### 3.B Relative ED tolerance level for each organ

**Figure 2:**
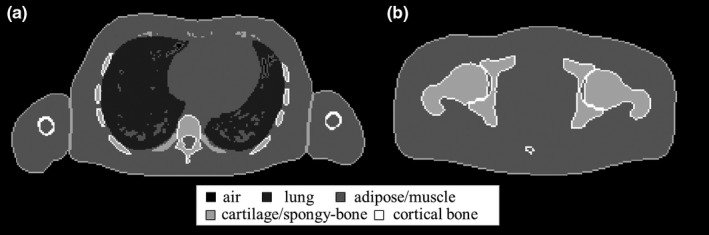
The adult reference computational phantom data in the International Commission on Radiological Protection publication 110 classified into five tissue groups. (a) Adult male chest plane which maximized lung thickness. (b) Adult male pelvic plane which maximized cartilage/spongy‐bone thickness.

Figure 2 shows axial planes of the lung and pelvis from ICRP‐110, and the maximum thicknesses of lung and cartilage/spongy‐bone tissues. Table 1 shows the maximum thickness and effective tissue thickness for each classified tissue group. Effective tissue thicknesses were defined considering the “worst case” in clinical situations when photon beams were used for 3DCRT. Therefore, the effective tissue thickness for lung and adipose/muscle tissues were round numbers between one‐half and two‐thirds of each maximum thickness, and the effective tissue thickness for cartilage/spongy‐bone, cortical bone, and tooth tissues were around the maximum thickness.

**Table 1:** The maximum thicknesses and effective tissue thicknesses measured by the adult reference computational phantom data in the International Commission on Radiological Protection publication 110

Classified tissue group	Male		Female		Effective tissue thickness for tolerance level (cm)
	Site	Maximum thickness (cm)	Site	Maximum thickness (cm)	
Lung	Chest	15.4	Chest	12.6	10
Adipose/Muscle	Pelvis	36.1	Pelvis	36.9	20
Cartilage/Spongy‐bone	Femora	9.8	Cranium	5.1	10
Cortical bone	–	0.9	–	1.1	1
Tooth	–	0.9	–	1.2	1

The tolerance levels of the CT‐ED calibration table were estimated with the corresponding ED tolerance curve of a 6 MVFFF beam and with the effective tissue thicknesses in Table 1. The tolerance levels of the 6 MVFFF beam for lung, adipose/muscle, and cartilage/spongy‐bone were 0.044, 0.022, and 0.044, respectively. Those tolerance levels denote that the relative dose error to local dose (Δ*D*/*D*) may reach 2%, which is caused by the CT‐ED calibration error. Regarding the cortical bone and tooth tissues, the effective tissue thicknesses were too small to establish a tolerance level.

### 3.C CT number constancy

The results of CT number constancy are summarized in Table 2. CTP682 was scanned with a CT scanner for routine quality assurance over a 20‐month period (n = 375). The standard deviation (SD) of the CT number was converted to the relative ED with the CT‐ED calibration table. All the 3 × SD of the relative ED were lower than 0.01 from the CT number of air to teflon.

**Table 2:** Summary of CT number consistency results for routine quality assurance over 20‐month period (n = 375). The SD of CT number was converted to relative ED with CT‐ED calibration table

	CT number		Relative ED	
	Mean	SD	SD	3 × SD
Air	−979.7	2.6	0.0026	0.0078
Lung #7112	−810.1	3.0	0.0030	0.0090
PMP	−182.5	1.7	0.0017	0.0052
LDPE	−92.3	2.4	0.0025	0.0074
Polystyrene	−36.3	3.1	0.0024	0.0071
Water	2.8	1.5	0.0012	0.0035
Acrylic	120.3	1.4	0.0004	0.0013
Bone 20%	237.8	2.6	0.0013	0.0039
Delrin	347.1	1.9	0.0009	0.0028
Bone 50%	697.5	4.9	0.0024	0.0073
Teflon	931.5	5.1	0.0025	0.0075

ED, electron density; SD, standard deviation; PMP, polymethylpentene; LDPE, low density polyethylene.

### 3.D Treatment planning with adult anthropomorphic phantoms

The typical photon beam treatment plan results are summarized in Table 3. The dose errors were caused by an increase equal to the relative ED tolerance levels of a 6 MVFFF beam, which were 0.044, 0.022 and 0.044 for lung, adipose/muscle and cartilage/spongy‐bone, respectively. The dose error of the 2 field (LR) plan for pelvic tumours was the largest of the errors in Table 3 because the effective depth was the longest compared to that of other plans. The impact with or without a flattening‐filter was about 0.2% of the prescribed dose. In any case, the dose errors caused by the CT‐ED calibration error were less than 2%, which was consistent with the tolerance level.

**Table 3:** Summary of the typical photon beam treatment plan results. Δ*D* and Δ*D*_m_ were dose errors caused by an increase amount equal to the tolerance levels of a 6 MVFFF beam

Tissue site	Energy	Delivery technique	Δ*D*/*D* at isocentre (%)	Δ*D*_m_/*D*_m_ in CTV (%)
Lung	6 MV	2 fields (AP)	−0.4	0.1
		8 fields	−0.7	−0.2
	6 MVFFF	2 fields (AP)	−0.6	−0.2
		8 fields	−0.9	−0.5
Prostate	10 MV	2 fields (LR)	−1.4	−1.4
		8 fields	−1.0	−1.0
	10 MVFFF	2 fields (LR)	−1.6	−1.5
		8 fields	−1.1	−1.0

FFF, flattening‐filter‐free; Δ*D*/*D*, the relative dose error to dose at isocentre; Δ*D*_m_/*D*_m_, the relative mean dose error to mean dose in CTV; CTV, clinical target volume; 3DCRT, three‐dimensional conformal radiotherapy; AP, anterior‐posterior; LR, left‐right.

## 4 DISCUSSION

The goal of this work was to establish a new relative ED tolerance level with a RTPS using an FFF beam because the photon spectrum of an FFF beam was softer than that of a flattened beam. In a previous work, Kilby et al.^2^ established the relative ED tolerance levels of a 6 MV beam for water, lung tissue, and bone tissue as ±0.03, ±0.05, and ±0.08, respectively. The tissue thicknesses (water: 20 cm, lung: 10 cm, bone: 7 cm) were defined by reviewing multiple treatment plans. Conversely, our work established the relative ED tolerance levels for both the 6 MV and 6 MVFFF beams. The relative ED tolerance levels of 6 MV for lung, adipose/muscle, and cartilage/spongy‐bone tissue were ±0.053, ±0.026, and ±0.053, respectively, and the relative ED tolerance levels of 6 MVFFF for lung, adipose/muscle, and cartilage/spongy‐bone tissue were ±0.044, ±0.022, and ±0.044, respectively. The CT number constancy was evaluated with routine quality assurance results. The impact of the CT number constancy was negligible because relative ED corresponding to the daily variance of the CT number was lower than 0.01. We defined the effective tissue thicknesses (lung: 10 cm, adipose/muscle: 20 cm, cartilage/spongy‐bone: 10 cm, cortical bone: 1 cm, tooth: 1 cm) by classifying the standard tissues of ICRP‐110 and by measuring the classified tissue group thicknesses. Because the classified tissue group was defined using whole‐body reference phantoms, the tolerance levels for cortical bone and tooth tissues were also estimated. Consequently, the thicknesses of cortical bone and tooth tissues were too small to define tolerance levels. In addition to these natural body components, man‐made materials may be implanted into the human body such as a hip, leg, and arm prostheses as well as spinal cord fixation devices and various dental fillings.^7^ However, these man‐made implants were beyond the scope of this work.

The tolerance levels were determined with a simple beam condition (10 × 10 cm field at a 10‐cm depth). Therefore, the dose error caused by the CT‐ED calibration error was simulated with an adult anthropomorphic phantom for a RTPS. In any case, the dose errors were less than 2%, which was consistent with tolerance levels. Although the impact with or without a flattening‐filter was about 0.2% of the prescribed dose, the tolerance levels should be determined by the lowest energy in useable beams for an RTPS.

The definition of relative ED tolerance levels was useful for the quality assurance of the CT‐ED calibration table of planning CT and cone beam CT. The CT‐ED calibration table was generally obtained using a calibration phantom with tissue substitutes. The CT‐ED calibration may slightly vary from that for calibration phantom types because of the phantom size and amount of solid water around the density inserts. Moreover, the CT‐ED calibration may vary from radiotherapy institutions because of the difference in the phantom production accuracy, tissue substitute choice and CT scan conditions. The relative ED tolerance levels were useful to approve CT‐ED calibration table for clinical use.

## 5 CONCLUSION

We have established the relative ED tolerance levels for each tissue type with an FFF beam. Because the tolerance levels are stricter when the beam energy decreases, the tolerance levels are determined by the lowest useable energy in a RTPS.

## CONFLICT OF INTEREST

The authors declare no conflict of interest.
